# Long-term outcome and cardiac function after anatomic repair of congenitally corrected transposition

**DOI:** 10.1093/icvts/ivae033

**Published:** 2024-03-05

**Authors:** Fumi Yokohama, Norihisa Toh, Yasuhiro Kotani, Yoichi Takaya, Yosuke Kuroko, Kenji Baba, Teiji Akagi, Shingo Kasahara, Hiroshi Ito

**Affiliations:** Department of Cardiovascular Medicine, Okayama University Graduate School of Medicine, Dentistry, and Pharmaceutical Sciences, Okayama, Japan; Department of Cardiovascular Medicine, Okayama University Graduate School of Medicine, Dentistry, and Pharmaceutical Sciences, Okayama, Japan; Department of Cardiovascular Surgery, Okayama University Graduate School of Medicine, Dentistry, and Pharmaceutical Sciences, Okayama, Japan; Department of Cardiovascular Medicine, Okayama University Graduate School of Medicine, Dentistry, and Pharmaceutical Sciences, Okayama, Japan; Department of Cardiovascular Surgery, Okayama University Graduate School of Medicine, Dentistry, and Pharmaceutical Sciences, Okayama, Japan; Department of Pediatric Cardiology, Okayama University Graduate School of Medicine, Dentistry and Pharmaceutical Sciences, Okayama, Japan; Department of Cardiovascular Medicine, Okayama University Graduate School of Medicine, Dentistry, and Pharmaceutical Sciences, Okayama, Japan; Department of Cardiovascular Surgery, Okayama University Graduate School of Medicine, Dentistry, and Pharmaceutical Sciences, Okayama, Japan; Department of Cardiovascular Medicine, Okayama University Graduate School of Medicine, Dentistry, and Pharmaceutical Sciences, Okayama, Japan

**Keywords:** Congenitally corrected transposition of the great arteries, Anatomic repair, Outcome

## Abstract

**OBJECTIVES:**

There is limited information on long-term outcomes and trajectories of ventricular and valvular functions in patients with congenitally corrected transposition of the great arteries after anatomic repair according to the operative strategy with a median follow-up period of more than 10 years.

**METHODS:**

Twenty-nine patients who underwent anatomic repair in Okayama University Hospital between January 1994 and December 2020 were reviewed. Outcomes were compared between patients who underwent a double switch operation (DS group) and patients with an atrial switch with a Rastelli operation (Rastelli–Senning/Mustard group).

**RESULTS:**

Fifteen (52%) were in the DS group and 14 (48%) were in the Rastelli–Senning/Mustard group. The median follow-up period after anatomic repair was 12.7 (interquartile range 4.2–18.8) years. There were 3 (10%) early deaths and 3 (10%) late deaths. Survival rates for the entire cohort at 10 and 20 years were 86% and 71%, respectively, and were not different between the 2 groups. Using competing risk analysis, risks of heart failure, cardiac rhythm device implantation and atrial arrhythmia showed no significant differences between the 2 groups, whereas risk of reoperation was higher in the Rastelli–Senning/Mustard group than that in the DS group. Four patients after a DS operation and 1 patient after a Rastelli technique developed more than moderate aortic regurgitation.

**CONCLUSIONS:**

During a median follow-up period of more than 10 years, mortality rate and ventricular and valvular functions after anatomic repair were acceptable, though the incidences of late complications were relatively high, especially in the Rastelli–Senning/Mustard group.

**Figure:**
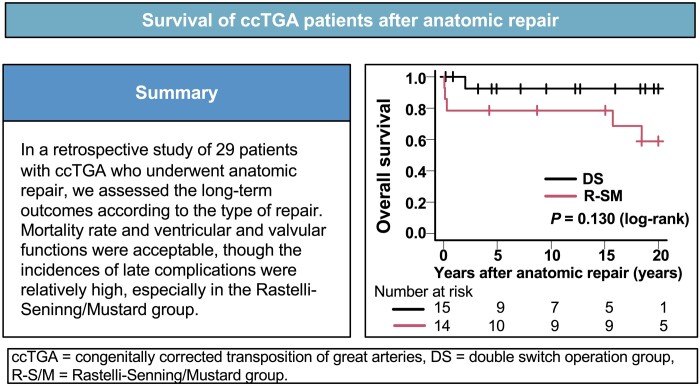

## INTRODUCTION

Anatomic repair has been widely performed for congenitally corrected transposition of the great arteries (ccTGA) and it is expected to enable maintenance of systemic ventricular and valvular functions. Although mid- and long-term outcomes have been shown in several studies [1–3], the median or mean follow-up durations were <10 years in most of those studies and comparisons of long-term outcomes between a double switch operation (DS group) and an atrial switch concomitant with a Rastelli operation have not been well investigated. Moreover, data on serial changes of ventricular and valvular functions are limited. The purpose of this study was to assess the long-term outcomes of anatomic repair in ccTGA patients according to the type of repair and to evaluate the detailed clinical course and serial changes of ventricular and valvular functions.

## PATIENTS AND METHODS

### Ethics statement

The Okayama University Hospital Institutional Review Board approved the study protocol (1712-002) and waived the need to obtain patient consent.

### Study population

We conducted a retrospective, single-centre study of patients with ccTGA who underwent anatomic repair in Okayama University Hospital. From January 1994 to December 2020, there were 55 patients with ccTGA who underwent surgical repair. Of those patients, patients who underwent physiologic repair (*n* = 6), univentricular repair (*n* = 15) and one-and-a-half ventricle repair (*n* = 5) were excluded. Therefore, the final study population included 29 patients with ccTGA after anatomic repair.

### Data collection and definitions

Surgical history, and clinical outcomes and data were obtained from the hospital database. Patients were divided into 2 groups: patients who underwent a DS group and patients who underwent a Senning or Mustard operation concomitant with a Rastelli operation (Rastelli–Senning/Mustard group) and we compared following outcomes between 2 groups. The primary outcome of interest was all-cause mortality. Early death was defined as death occurring before hospital discharge or within 30 days after anatomic repair. We also analysed cumulative event rates for heart failure, device implantation, atrial tachyarrhythmia and surgical reintervention after anatomic repair. Ventricular ejection fraction (EF) was evaluated by echocardiography by using modified Simpson method and we used correlations of normal for EF ≥50%, mildly reduced for EF of 40–49% and moderately reduced for EF of 30–39%.

### Statistical analysis

All of the data were analysed using IBM SPSS Statistics version 24.0 (IBM-SPSS Inc, Armonk, NY). The normality of continuous variables was assessed using Shapiro–Wilk test. Continuous variables were reported as medians and interquartile ranges (IQRs) and were examined using the Mann–Whitney *U*-test. Categorical variables were examined using the chi-squared test. Patient mortality was assessed using Kaplan–Meier curves and log-rank test was used to assess the association the type of procedure with mortality. The assumption of proportional hazards was checked in all cases. Survival and freedom from death, heart failure, device therapy, atrial tachyarrhythmia or surgical reintervention were presented from a competing risk analysis. Cox proportional hazards regression models were built to identify predictors of late survival and late left ventricular (LV) dysfunction.

## RESULTS

### Patients’ characteristics and anatomy

A total of 29 patients who underwent an anatomic repair were included in the present study. Fifteen of those 29 patients underwent a DS group and 14 patients underwent a Senning or Mustard operation concomitant with a Rastelli operation (Rastelli–Senning/Mustard group). Characteristics of the patients are shown in Table 1.

**Table 1:** Patient demographics, morphologies and palliation characteristics

	Total (*n* = 29)	DS (*n* = 15)	Rastelli–Senning/Mustard (*n* = 14)	*P*-value
Demographics				
** **Male	21 (72)	13 (87)	8 (57)	0.086
** **Age at definitive surgery (years)	4.9 (3.2–7.3)	3.4 (2.6–4.8)	6.0 (5.0–8.7)	0.010
Morphology				
** **Dextrocardia	9 (31)	2 (13)	7 (50)	0.017
** **Situs inversus	4 (14)	0	4 (29)	0.042
** **VSD	22 (76)	8 (53)	14 (100)	0.004
** **PS/Pulmonary atresia	14 (48)	0	14 (100)	<0.001
** **Ebstein’s anomaly of TV	3 (10)	3 (20)	0	0.125
Rhythm disorder				
** **Complete atrioventricular block	2 (7)	2 (13)	0	0.259
Palliation				
** **PAB	14 (48)	14 (93)	0	<0.001
** **Systemic-to-pulmonary shunt	10 (34)	0	10 (71)	<0.001
** **Median age at palliation (years)	0.5 (0.1–1.8)	1.0 (0.4–2.7)	0.2 (0.1–0.3)	0.031
** **Median duration of palliation (years)	4.0 (2.3–5.8)	2.5 (1.7–3.2)	5.8 (5.0–7.1)	<0.001

Data are presented as number (%) or median (interquartile range).

DS: double switch operation; PAB: pulmonary artery banding; PS: pulmonary stenosis; TV: tricuspid valve; VSD: ventricular septal defect.

### Palliative surgeries before anatomic repair

Details of surgical palliations are shown in Table 1. Fourteen patients (48%) underwent pulmonary artery banding (PAB). The median age at the 1st PAB was 1.0 years (IQR 0.4–2.7 years) and the median time interval from the 1st PAB to anatomic repair was 2.5 years (IQR 1.7–3.2 years). Three of those 14 patients required re-PAB before anatomic repair.

Ten patients (34%) underwent systemic-to-pulmonary shunt placement. The median age at the 1st systemic-to-pulmonary shunt placement was 0.2 years (IQR 0.1–0.3 years) and the median time interval from the 1st palliative surgery to anatomic repair was 5.8 years (IQR 5.0–7.1 years). Seven of those 10 patients required multiple palliative shunt procedures.

### Anatomic repair and concomitant operation

Primary anatomic repairs were achieved in 5 (17%) of the patients, including 1 patient in the DS group and 4 patients in the Rastelli–Senning/Mustard group. The age at the time of anatomic repair was older in the Rastelli–Senning/Mustard group than in the DS group (*P *=* *0.010) (Table 1). Concomitant operations performed during anatomic repair are shown in Table 2.

**Table 2:** Perioperative and postoperative characteristics

	Total (*n* = 29)	DS (*n* = 15)	Rastelli–Senning/Mustard (*n* = 14)	*P*-value
Type of atrial switch operation, *n* (%)				
** **Senning operation	28 (97)	15 (100)	13 (93)	0.483
** **Mustard operation	1 (3)	0	1 (7)	0.483
Concomitant procedure, *n* (%)				
** **VSD closure or intracardiac rerouting	21 (72)	7 (47)	14 (100)	0.001
** **TV plasty	2 (7)	2 (13)	0	0.259
** **Peripheral PA plasty	4 (14)	1 (7)	3 (21)	0.143
Preoperative ventricular and valvular function, *n*				
** **LV function (normal/mild dysfunction/moderate dysfunction)	28/1/0	14/1/0	14/0/0	0.326
** **RV function (normal/mild dysfunction/moderate dysfunction)	29/0/0	15/0/0	14/0/0	NA
** **MR (mild/moderate/severe)	3/0/0	0/0/0	3/0/0	0.058
** **TR (mild/moderate/severe/status post TV plasty)	15/7/2/1	7/6/1/1	8/1/1/0	0.072
** **Native PR (mild/moderate/severe)	2/0/0	2/0/0	0/0/0	0.157
Postoperative ventricular and valvular function, n	(*n* = 26)	(*n* = 15)	(*n* = 11)	
** **LV function (normal/mild dysfunction/moderate dysfunction)	25/0/1	14/0/1	11/0/0	0.382
** **RV function (normal/mild dysfunction/moderate dysfunction)	26/0/0	15/0/0	11/0/0	NA
** **MR (mild/moderate/severe)	13/0/0	9/0/0	4/0/0	0.234
** **TR (mild/moderate/severe/status post TV plasty)	14/1/0/3	7/0/0/3	7/1/0/0	0.264
** **Neo-AR/AR (mild/moderate/severe)	11/1/1	8/1/1	3/0/0	0.216

Data are presented as number (%), mean ± standard deviation, or median (interquartile range).

AR: aortic regurgitation; DS: double switch operation; LV: left ventricular; MR: mitral regurgitation; PA: pulmonary artery; PR: pulmonary regurgitation; RV: right ventricular; TR: tricuspid regurgitation; TV: tricuspid valve; VSD: ventricular septal defect; NA: not applicable.

Preoperatively, 28 patients (97%) had normal LV ejection fraction and all of the patients had normal right ventricular (RV) ejection fraction (Table 2). Nine patients (31%) had moderate or greater tricuspid regurgitation and 1 patient had undergone tricuspid valve plasty prior to anatomic repair.

### Follow-up and overall survival

Follow-up data were complete for all patients. There were 6 deaths: 3 early deaths (all in the Rastelli–Senning/Mustard group) and 3 late deaths (1 in the DS group and 2 in the Rastelli–Senning/Mustard group) (Table 3). The median follow-up periods after anatomic repair were 12.7 years (IQR 4.2–18.8 years).

**Table 3:** Postoperative outcomes by group

	Total (*n* = 29)	DS (*n* = 15)	Rastelli–Senning/Mustard (*n* = 14)	*P*-value
Death	6	1	5	0.070
** **Early death	3	0	3	0.100
** **Late death	3	1	2	0.381
Heart failure	4	3	1	0.426
Cardiac rhythm device implantation after anatomic repair	6	5	1	0.165
** **Permanent pacemaker implantation for SSS	3	3	0	0.175
** **Permanent pacemaker implantation for AVB	2	1	1	0.667
** **CRT implantation for LV dysfunction	1	1	0	0.577
Atrial tachyarrhythmia	6	2	4	0.183
** **Paroxysmal supraventricular tachycardia	3	0	3	0.063
** **Paroxysmal atrial tachycardia	3	2	1	0.619
Surgical reintervention after anatomic repair (number of patients)	11	3	8	0.010
Type of surgical reintervention (number of operations)				
** **RVOT reconstruction	7	0	7	NA
** **Relief of PVO	3	1	2	NA
** **Baffle leak closure	2	0	2	NA
** **Peripheral pulmonary artery reconstruction	2	1	1	NA
** **Neo-aortic valve plasty or replacement	3	3	0	NA
** **SVC enlargement	1	1	0	NA
** **Coronary artery plasty	1	1	0	NA
** **TV plasty	1	0	1	NA
Ventricular and valvular function at the latest visit, n	(n = 23)	(n = 14)	(n = 9)	
** **LV function (normal/mild dysfunction/moderate dysfunction)	22/1/0	13/1/0	9/0/0	0.412
** **RV function (normal/mild dysfunction/moderate dysfunction)	23/0/0	14/0/0	9/0/0	NA
** **MR (mild/moderate/severe)	12/1/0	8/1/0	4/0/0	0.520
** **TR (mild/moderate/severe/status post TV plasty)	13/2/0/3	8/1/0/2	5/1/0/1	0.986
** **Neo-AR/AR (mild/moderate/severe/status post AV plasty)	11/3/0/1	7/2/0/1	4/1/0/0	0.773

Data are presented as number.

AR: aortic regurgitation; AV: aortic valve; AVB: atrioventricular block; CRT: cardiac resynchronization therapy; DS: double switch operation; LV: left ventricular; MR: mitral regurgitation; PVO: pulmonary venous pathway obstruction; RV: right ventricular; RVOT: right ventricular outflow tract; SSS: sick sinus syndrome; SVC: superior vena cava; TR: tricuspid regurgitation; TV: tricuspid valve; NA: not applicable.

**Figure 1:**
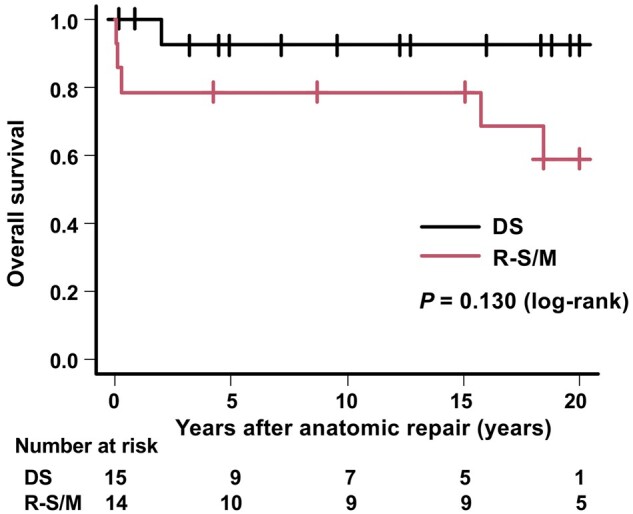
Kaplan–Meier and log-rank analyses for overall survival. In the DS group, survival rates were 92% and 92% at 10 and 20 years after anatomic, respectively. In the Rastelli–Senning/Mustard group, the numbers were 79% and 59%, respectively. DS: double switch; R-S/M: Rastelli–Senning/Mustard.

Survival rates after anatomic repair at 1, 10 and 20 years were 90%, 86% and 71%, respectively. There was no significant difference in the survival rates between the DS group and the Rastelli–Senning/Mustard group (100%, 92% and 92% in the DS group vs 79%, 79% and 59% in the Rastelli–Senning/Mustard group at 1, 10 and 20 years, respectively, *P *=* *0.130) (Fig. 1).

### Early death

The early deaths occurred 27, 33 and 104 days after anatomic repair (Table 3). The patient who died 27 days after anatomic repair underwent a Rastelli and Senning operation at the age of 5 years and died due to postoperative wound infection. The patient who died 33 days after anatomic repair underwent a Rastelli and Senning operation at the age of 5 years and died due to postoperative wound infection. The patient who died 104 days after anatomic repair underwent a Rastelli and Senning operation at the age of 29 years and died due to ventricular tachycardia. In univariate analysis, only situs inversus was related to early death (*P *=* *0.035).

### Late death

The late deaths occurred 2, 15 and 18 years after anatomic repair. The patient who died 2 years after anatomic repair had undergone a DS group at the age of 11 years after multiple PABs. Seven months after the operation, the patient underwent mechanical aortic valve replacement due to severe neo-aortic regurgitation (neo-AR) and moderate LV dysfunction. One year after the aortic valve replacement, a repeat aortic valve replacement was performed due to mechanical valve thrombosis, but the patient died of severe LV dysfunction at the age of 13 years. The patient who died 15 years after anatomic repair had undergone a Rastelli and Senning operation at the age of 6 years and died suddenly at the age of 22 years. The patient who died 18 years after anatomic repair had undergone a Rastelli and Senning operation at the age of 16 years. The patient died due to out-of-hospital cardiac arrest at the age of 35 years. The latter 2 patients had had regular clinical follow-up, and ventricular dysfunction, structural abnormalities and rhythm disturbance had not been documented. In univariate analysis, any data were not related to late death.

### Secondary adverse events

**Figure 2:**
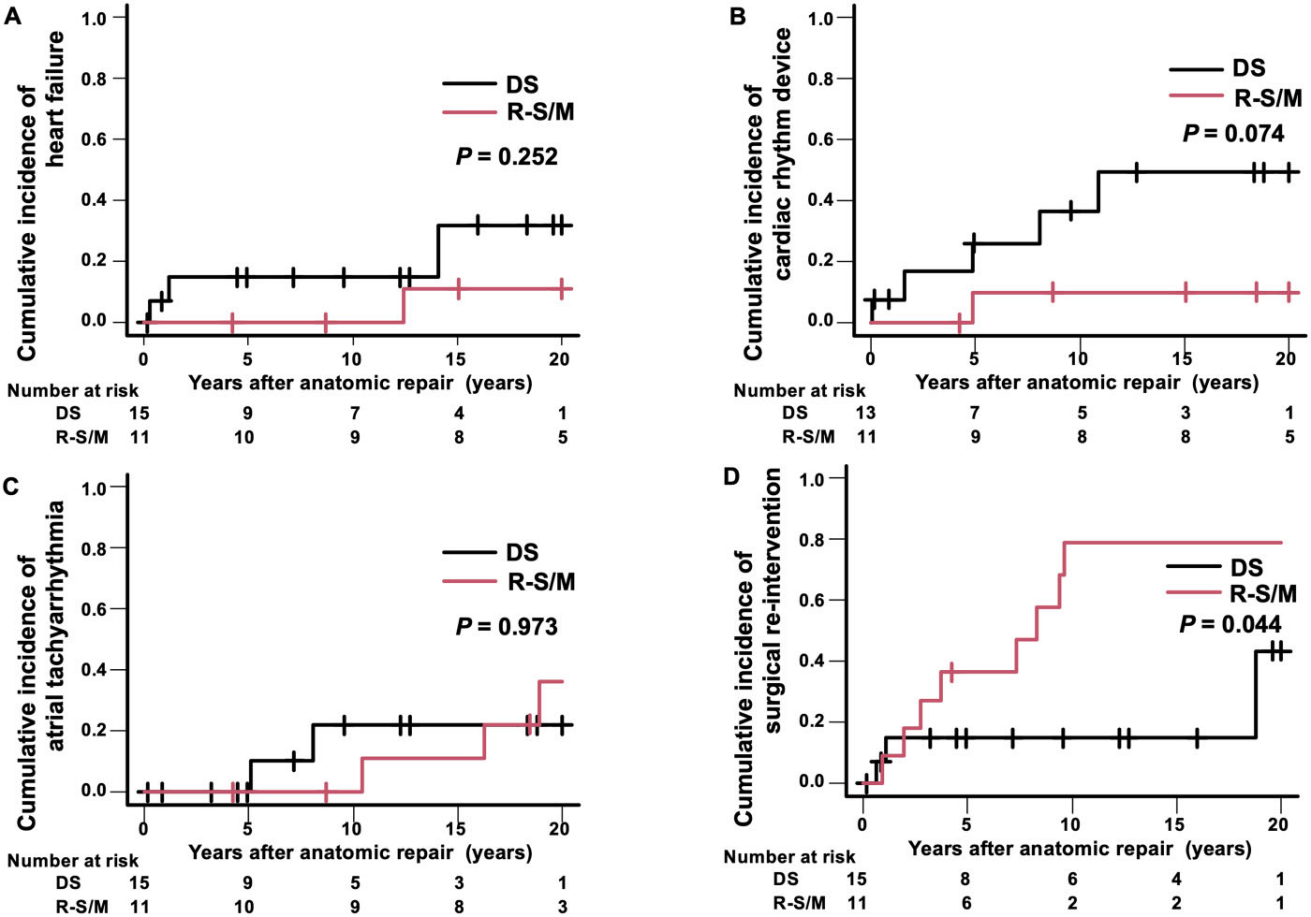
Competing risk analysis of events following the anatomic repair. (**A**) Heart failure. (**B**) Cardiac rhythm device implantation. (**C**) Atrial arrhythmia. (**D**) Reoperation. DS: double switch; R-S/M: Rastelli–Senning/Mustard.

During the follow-up period, 4 patients (3 in the DS group and 1 in the Rastelli–Senning/Mustard group) developed heart failure (Table 3). Competing risk analysis predicted that after 10 years from anatomic repair, 0% had died without heart failure, 15% had heart failure and 85% remained alive without heart failure in the DS group, and the numbers were 0%, 0% and 100%, respectively in the Rastelli–Senning/Mustard group (*P *=* *0.252) (Fig. 2A).

Two patients underwent permanent pacemaker implantation before and during anatomic repair. During the follow-up period, 6 patients (5 in the DS group and 1 in the Rastelli–Senning/Mustard group) had device therapy after anatomic repair (Table 3). Five patients (2 with atrioventricular block and 3 with sick sinus syndrome) had permanent pacemaker implantation and 1 patient in the DS group received cardiac resynchronization therapy for systemic LV dysfunction. Competing risk analysis predicted that after 10 years from anatomic repair, 0% had died without cardiac rhythm device implantation, 37% underwent cardiac rhythm device implantation and 63% remained alive without cardiac rhythm device implantation in the DS group, and the numbers were 0%, 10% and 90%, respectively, in the Rastelli–Senning/Mustard group (*P *=* *0.074) (Fig. 2B).

In the present cohort, ventricular tachyarrhythmia was documented in 1 patient who died in the early postoperative period. Atrial tachyarrhythmia occurred in 6 patients after anatomic repair (3 with paroxysmal supraventricular tachycardia and 3 with paroxysmal atrial tachycardia) (Table 3). Five of those 6 patients received radiofrequency catheter ablation after atrial septal puncture and tachyarrhythmias were successfully controlled with or without anti-arrhythmic drugs in all of those patients. Competing risk analysis predicted that after 10 years from anatomic repair, 8% had died without atrial arrhythmia, 22% had atrial arrhythmia and 70% remained alive without atrial arrhythmia in the DS group and the numbers were 0%, 0% and 100%, respectively, in the Rastelli–Senning/Mustard group (*P *=* *0.973) (Fig. 2C).

During the follow-up period, 11 patients (3 in the DS group and 8 in the Rastelli–Senning/Mustard group) underwent a total of 20 surgical re-interventions following anatomic repair (Table 3). Competing risk analysis predicted that after 10 years from anatomic repair, 0% had died without reoperation, 15% underwent reoperation and 85% remained alive without reoperation in the DS group, and the numbers were 0%, 79% and 21%, respectively, in the Rastelli–Senning/Mustard group (*P *=* *0.044) (Fig. 2D).

### Ventricular and valvular functions before and after anatomic repair

**Figure 3:**
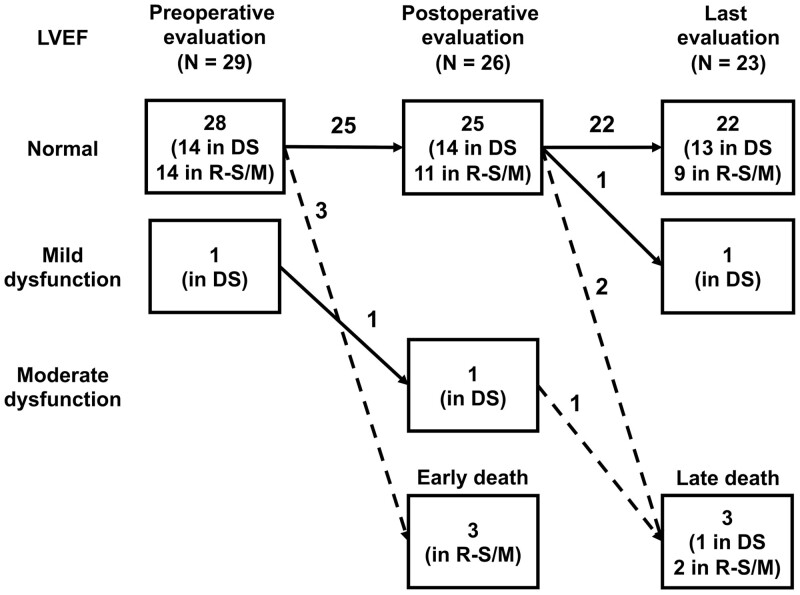
Serial changes of LVEF during the follow-up period. DS: double switch; LVEF: left ventricular ejection fraction; R-S/M: Rastelli–Senning/Mustard.

**Figure 4:**
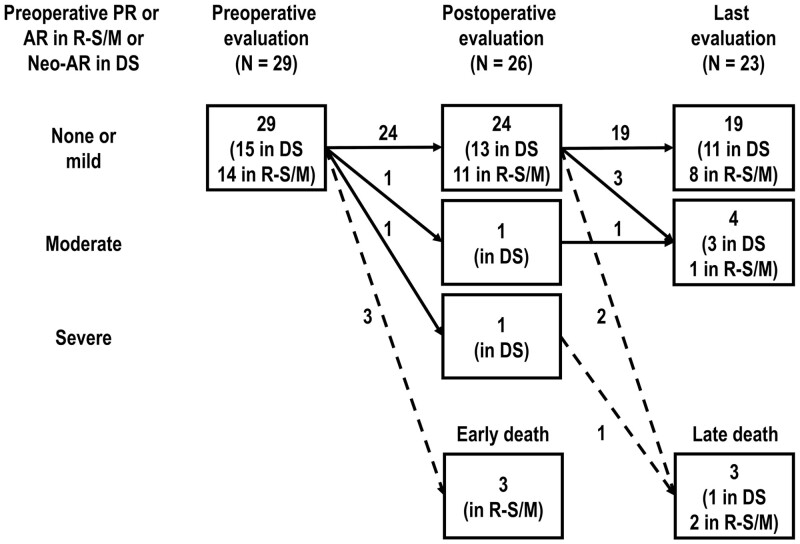
Serial changes of neo-AR/AR severity during the follow-up period. AR: aortic regurgitation; DS: double switch; PR: pulmonary regurgitation; R-S/M: Rastelli–Senning/Mustard.

Longitudinal data for ventricular and valvular functions before and after anatomic repair are shown in Tables 2 and 3 and Figs 3 and 4. All of the 3 patients who died in the early postoperative period had normal LV function in the preoperative and postoperative evaluations. At the latest follow-up, 22 (96%) of the 23 late survivors had normal LV ejection fraction (Fig. 3). In the present study, 2 patients developed LV dysfunction during the study period. The 1 patient developed moderate LV dysfunction due to severe neo-AR after the DS group. The other patient developed mild LV dysfunction without an apparent cause late after the DS group.

PAB was performed for the following reasons; 11 patients for LV training, 3 patients for heart failure treatment, and 1 patient without detailed information. Univariable Cox regression testing revealed that PAB was not associated with late LV dysfunction [odds ratio <0.001 (95% CI 0–infinite), *P *=* *1.00].

In the DS group, 11 of the 15 patients did not develop more than moderate neo-AR throughout the entire observational period (Fig. 4). On the other hand, only 1 (7%) of the 14 patients in the Rastelli–Senning/Mustard group developed moderate AR during the entire follow-up period. None of the patients had more than mild mitral regurgitation before the anatomic repair. Only 1 patient in the DS group had moderate mitral regurgitation at the latest follow-up. No patient underwent mitral valve surgery during the observational period.

## Discussion

This study showed long-term mortality and detailed clinical outcomes and trajectories of ventricular and valvular functions during a median follow-up period of 12 years after anatomic repair in ccTGA patients.

Although anatomic repair has been expected to improve long-term systemic ventricular function and prognosis, relatively high rate of early mortality with ranging up to 10% and progressive LV dysfunction have been reported [1–6] because of complexity of the operation, prolonged aortic clamping time [7], maladaptation of the LV to afterload of systemic circulation [8] and complication of neo-AR [1, 6]. In our cohort, although 3 patients (10%) died in the early postoperative period, the long-term survival rate was not inferior to the rates in previous studies [6, 9]. Moreover, the median follow-up period in the present study was much longer than the following periods in previous studies and thus the current study strengthens the reasonable long-term prognosis of anatomic repair.

The major causes of death after anatomic repair are LV dysfunction followed by heart failure and sudden death [1–3, 8]. LV dysfunction is not uncommon late after anatomic repair [2], and in the current study 2 of the 26 early survivors developed LV dysfunction. There are several possible causes of death and LV dysfunction in the remote period, including neo-AR, PAB, ventricular pacing and atrial tachyarrhythmia.

Neo-AR could develop at any stage after anatomic repair with the deterioration of the original pulmonary valve [10]. In our cohort, progressive neo-AR was observed late after anatomic repair, being consistent with previous reports [1–3, 6, 8]. PAB was reported to be associated with late neo-AR in previous reports [11]. In the present study, PAB was performed in most cases (14 of 15 DS patients), and data on preoperative pulmonary arterial diameter that may affect neo-AR were limited. Therefore, we could not investigate the association between PAB and early and late postoperative neo-AR. And although PAB for LV retraining is previously reported as a strong predictor of late LV dysfunction [12], the association between PAB and late LV dysfunction could not be demonstrated in our cohort. Ventricular pacing is also associated with LV dysfunction, which may be improved by biventricular pacing even in ccTGA patients after anatomic repair [1, 2]. However, we could not discuss the impact of biventricular pacing because biventricular pacing was performed in only 1 patient. Regarding atrial tachyarrhythmias, catheter ablation and anti-arrhythmic drugs were effective in our cohort, whereas difficulties in treating atrial arrhythmias and maintaining sinus rhythm were described in previous reports [8]. In the Rastelli–Senning/Mustard group, although RV incision was reported to be associated with RV dysfunction [13], long-term RV function was preserved in all patients, and ventricular tachyarrhythmia was observed only in 1 patient early after the operation. However, in the Rastelli–Senning/Mustard group, the high frequency of conduit replacement led to a greater number of reoperations compared to that in the DS group.

The actuarial incidence rate of sudden cardiac death in ccTGA patients after anatomic repair is unknown, although sudden death late has been reported in several cohorts [1–3]. Complete atrioventricular block, which is a well-known risk factor of sudden death, is frequently observed after anatomic repair [4, 14]. However, in the present study, sudden death was observed in 2 early survivors although fatal arrhythmias were not detected in Holter monitoring performed every 6 months to a year.

In our institution, anatomic repair has been performed based on our previous report [15]: (i) near-systemic pressure in a well-functioning morphological LV (LV pressure/RV pressure ratio >0.75); (ii) a sufficient rise in LV pressure in response to catecholamine stimulation; and (iii) an adequate LV hypertrophy (LV mass index >80 g/m^2^). In the current study, the number of patients after physiologic repair was small since we performed anatomic repair with the expectation that systemic LV will improve the long-term prognosis. Moreover, 2 of 6 patients underwent physiologic repair after reaching adulthood. Therefore, we did not compare the outcomes between anatomic and physiologic repair in the present study.

### Limitations

There are some limitations in this study. First, since ccTGA is a rare form of congenital heart disease and the number of patients in the present study was small, the number of patients in our cohort was not sufficient to represent all anatomically repaired ccTGA survivors and draw robust conclusions. Moreover, due to the limited number of study subjects and inadequate statistical power, a type II error could not be avoided. Second, the surgical indications differ between the DS operation and Rastelli–Senning/Mustard operation, and thus the results of the present study are unlikely to have a significant impact on the choice of surgical procedure. However, the present study showed that the long-term LV function after LV training in the DS group was not inferior to that in the Rastelli–Senning/Mustard group. Furthermore, the long-term RV function in the Rastelli–Senning/Mustard group was not inferior to that in the DS group even after the RV incision. Third, not all of the data for arrhythmic events were obtained because arrhythmic data were reviewed from medical records. Fourth, the decision for surgical reintervention was made on the basis of a single-centre policy. Fifth, although serial changes of postoperative electrocardiogram may be helpful for predicting conduction abnormality after the surgery, these data were limited and thus further investigations are warranted. Sixth, the study period is very long and the patient inclusion rate per year is low. Therefore, although our institutional strategy for anatomic repair is consistent in the study period, we could not completely eliminate the effect of changes of clinical practice throughout a study period on the heterogeneity of the findings.

## CONCLUSIONS

With more than 12 years of follow-up, long-term survival and LV and valvular functions late after anatomic repair were acceptable irrespective of the type of surgical procedure, although the incidence of late complications was relatively high.

## Data Availability

The data will be shared on reasonable request.

## ABBREVIATIONS

AR: Aortic regurgitation
ccTGA: Congenitally corrected transposition of the great arteries
DS: Double switch operation
EF: Ejection fraction
IQR: Interquartile range
LV: Left ventricular
PAB: Pulmonary artery banding
RV: Right ventricular

## References

[1] Murtuza B , Barron DJ, Stumper O, Stickley J, Eaton D, Jones TJ et al Anatomic repair for congenitally corrected transposition of the great arteries: a single-institution 19-year experience. J Thorac Cardiovasc Surg 2011;142:1348–57.e1. 21955471 10.1016/j.jtcvs.2011.08.016

[2] Bautista-Hernandez V , Myers PO, Cecchin F, Marx GR, Del Nido PJ. Late left ventricular dysfunction after anatomic repair of congenitally corrected transposition of the great arteries. J Thorac Cardiovasc Surg 2014;148:254–8. 24100093 10.1016/j.jtcvs.2013.08.047

[3] Hraska V , Vergnat M, Zartner P, Hart C, Suchowerskyj P, Bierbach B et al Promising outcome of anatomic correction of corrected transposition of the great arteries. Ann Thorac Surg 2017;104:650–6. 28648534 10.1016/j.athoracsur.2017.04.050

[4] Lenoir M , Bouhout I, Gaudin R, Raisky O, Vouhé P. Outcomes of the anatomical repair in patients with congenitally corrected transposition of the great arteries: lessons learned in a high-volume centre. Eur J Cardiothorac Surg 2018;54:532–8. 29566142 10.1093/ejcts/ezy116

[5] Myers PO , del Nido PJ, Geva T, Bautista-Hernandez V, Chen P, Mayer JE Jr et al Impact of age and duration of banding on left ventricular preparation before anatomic repair for congenitally corrected transposition of the great arteries. Ann Thorac Surg 2013;96:603–10. 23820627 10.1016/j.athoracsur.2013.03.096

[6] Brizard CP , Lee A, Zannino D, Davis AM, Fricke TA, d’Udekem Y et al Long-term results of anatomic correction for congenitally corrected transposition of the great arteries: a 19-year experience. J Thorac Cardiovasc Surg 2017;154:256–65.e4. 28476422 10.1016/j.jtcvs.2017.03.072

[7] Shin’oka T , Kurosawa H, Imai Y, Aoki M, Ishiyama M, Sakamoto T et al Outcomes of definitive surgical repair for congenitally corrected transposition of the great arteries or double outlet right ventricle with discordant atrioventricular connections: risk analyses in 189 patients. J Thorac Cardiovasc Surg 2007;133:1318–28. 17467450 10.1016/j.jtcvs.2006.11.063

[8] Sharma R , Talwar S, Marwah A, Shah S, Maheshwari S, Suresh P et al Anatomic repair for congenitally corrected transposition of the great arteries. J Thorac Cardiovasc Surg 2009;137:404–12.e4. 19185160 10.1016/j.jtcvs.2008.09.048

[9] Barrios PA , Zia A, Pettersson G, Najm HK, Rajeswaran J, Bhimani S et al; Members of the ccTGA Working Group. Outcomes of treatment pathways in 240 patients with congenitally corrected transposition of great arteries. J Thorac Cardiovasc Surg 2021;161:1080–93.e4. 33436290 10.1016/j.jtcvs.2020.11.164

[10] Hurle JM , Colvee E. Changes in the endothelial morphology of the developing semilunar heart valves. A TEM and SEM study in the chick. Anat Embryol (Berl) 1983;167:67–83. 6881544 10.1007/BF00304601

[11] McMahon CJ , Ravekes WJ, Smith EO, Denfield SW, Pignatelli RH, Altman CA et al Risk factors for neo-aortic root enlargement and aortic regurgitation following arterial switch operation. Pediatr Cardiol 2004;25:329–35. 14727099 10.1007/s00246-003-0483-6

[12] Quinn DW , McGuirk SP, Metha C, Nightingale P, de Giovanni JV, Dhillon R et al The morphologic left ventricle that requires training by means of pulmonary artery banding before the double-switch procedure for congenitally corrected transposition of the great arteries is at risk of late dysfunction. J Thorac Cardiovasc Surg 2008;135:1137–44. 18455595 10.1016/j.jtcvs.2008.02.017

[13] Egbe AC , Miranda WR, Stephens EH, Anderson JH, Andi K, Goda A et al Right ventricular systolic dysfunction in adults with anatomic repair of d-transposition of great arteries. Am J Cardiol 2023;192:101–8. 36764091 10.1016/j.amjcard.2023.01.025 PMC10402278

[14] Furuya T , Hoashi T, Shimada M, Imai K, Komori M, Kurosaki K et al Serial changes of tricuspid regurgitation after anatomic repair for congenitally corrected transposition. Eur J Cardiothorac Surg 2020;58:163–70. 32048707 10.1093/ejcts/ezaa022

[15] Honjo O , Kawada M, Akagi T, Kotani Y, Ishino K, Sano S. Left ventricular retraining and anatomic correction in teenage patient with congenitally corrected transposition of the great arteries. Circ J 2007;71:613–6. 17384468 10.1253/circj.71.613

